# Supplementary material for: Wafer-Scale All-Dielectric Quasi-BIC Metasurfaces: Bridging High-Throughput Deep-UV Lithography with Nanophotonic Applications
Source: Nano Lett. 2026 Feb 6;26(6):2059–67. doi: 10.1021/acs.nanolett.5c05226 (PMC12922174; doi:10.1021/acs.nanolett.5c05226)
Supplement: Supplementary file 1 [file nl5c05226_si_001.pdf]

Supporting Information for

Wafer-Scale All-Dielectric quasi-BIC Metasurfaces: Bridging High-throughput Deep-UV Lithography with Nanophotonic Applications

Aidana Beisenova[1], Wihan Adi[1], Wenxin Wu[2], Shovasis K Biswas[2], Samir Rosas[1], Biljana Stamenic[3], Demis D. John[3], Filiz Yesilkoy[1,*]

[1] Department of Biomedical Engineering, University of Wisconsin-Madison, Madison, WI 53706, USA

[2] Department of Electrical and Computer Engineering, University of Wisconsin-Madison, Madison, WI 53706, USA

[3] Nanofabrication Facility, Department of Electrical and Computer Engineering, University of California, Santa Barbara, CA 93106, USA

*Corresponding author email: filiz.yesilkoy@wisc.edu

Note 1. Double-hole structure simulations for different cases

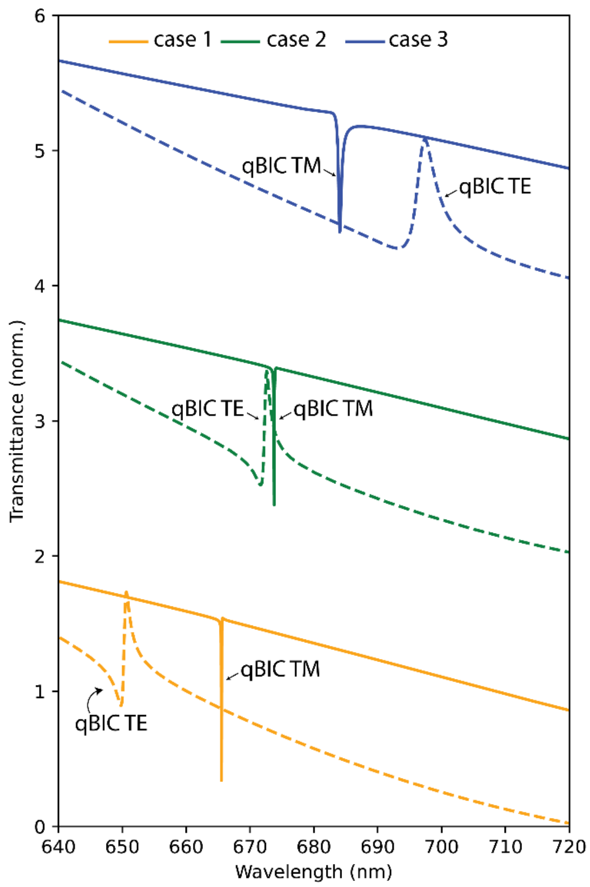

Figure S1. Simulated transmission spectra of the double-hole structure with different radius values for each case. Simulation in case 1 (yellow) has $r_1 = 125$ nm, $r_2 = 100$ nm. Simulation in case 2 (green) has $r_1 = 125$ nm, $r_2 = 70$ nm. Simulation in case 3 (blue) has $r1 = 135$nm, $r2 = 110$ nm.

Note 2. Cross-sectional height from AFM measurements

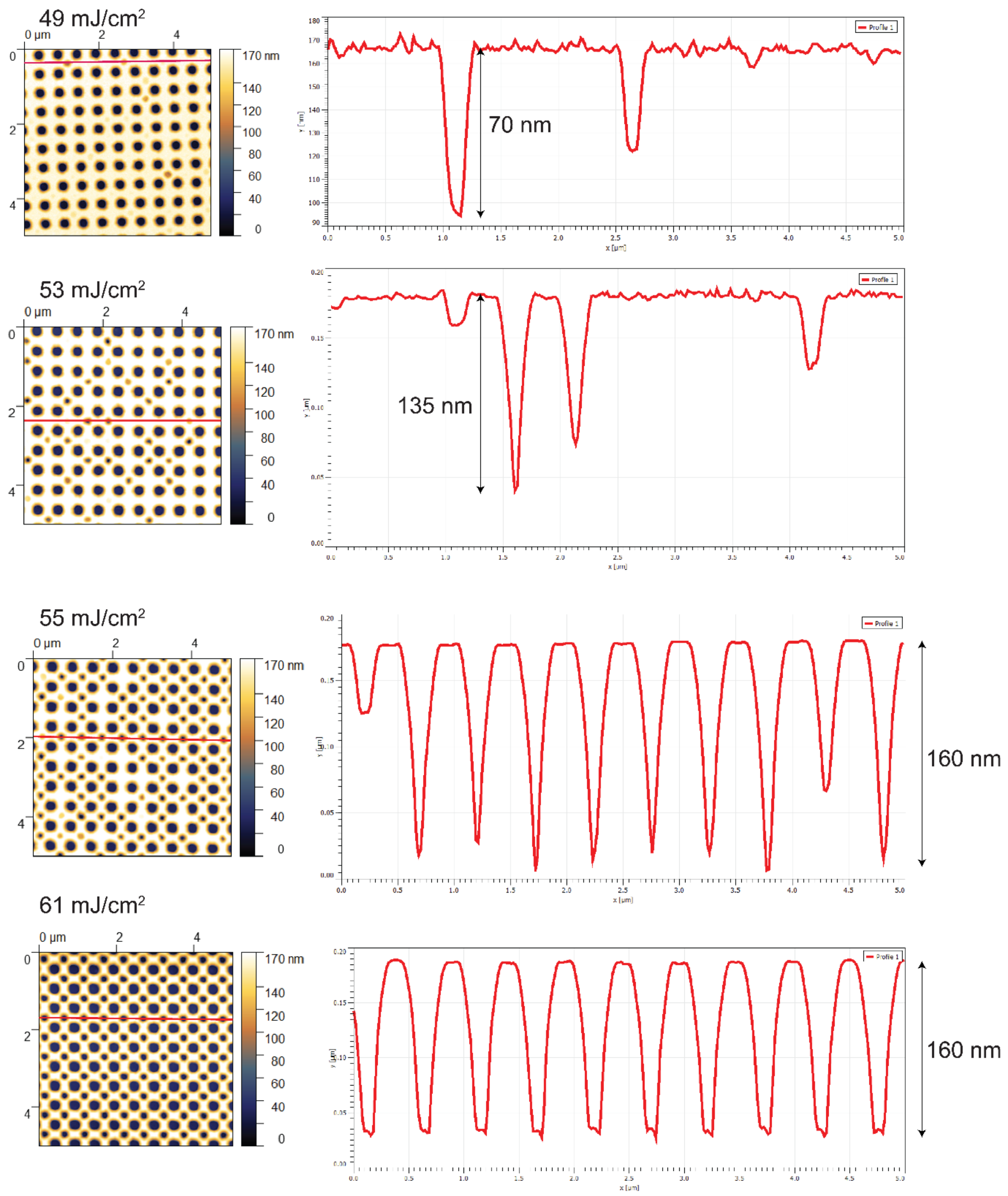

Figure S2. Atomic force microscopy (AFM) images and their corresponding cross-sectional height profiles at the central small hole positions (along the red lines) from 49 mJ/cm^2 to 61 mJ/cm^2. Pointy depth profile indicates a lower bound of the hole depth due to difficulty in measuring trenches with high aspect ratio.

Note 3. Supercell simulations

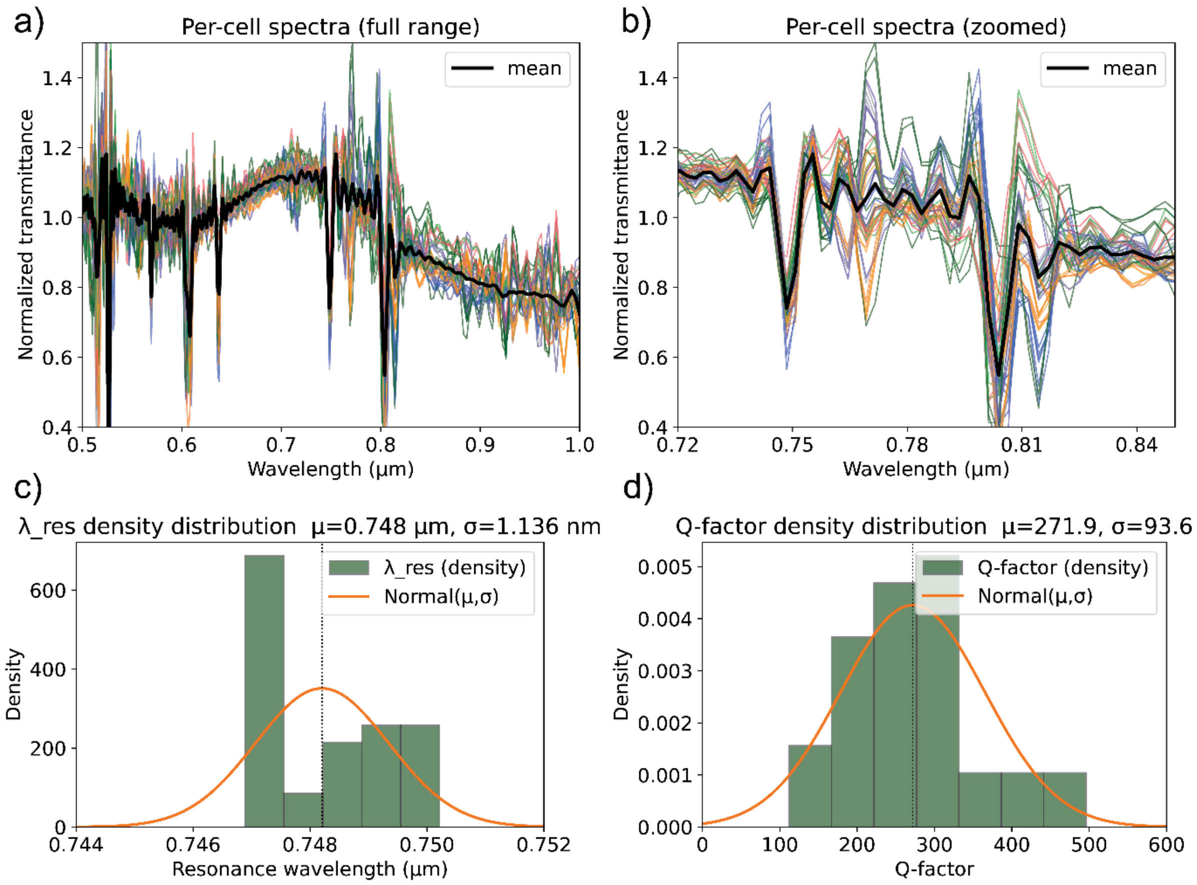

Figure S3. (a) Normalized hyperspectral transmittance spectra of a 10×10 supercell simulated with periodic boundary conditions, where the smaller hole depth in each unit cell is varied (μ of the depth = 40 nm, σ = 25 nm). The black curve indicates the mean spectrum across all cells. **(b)** Zoomed-in view of the spectra around the quasi-bound state in the continuum (qBIC) resonances. **(c)** Distribution of the extracted resonance wavelengths (λ_{res}) of the qBIC TE mode, centered around λ_{res} ≈ 750 nm. **(d)** Corresponding Q-factor distribution with fitted Gaussian profiles shown in orange.

Note 4. Metasurface measurements in a dry condition

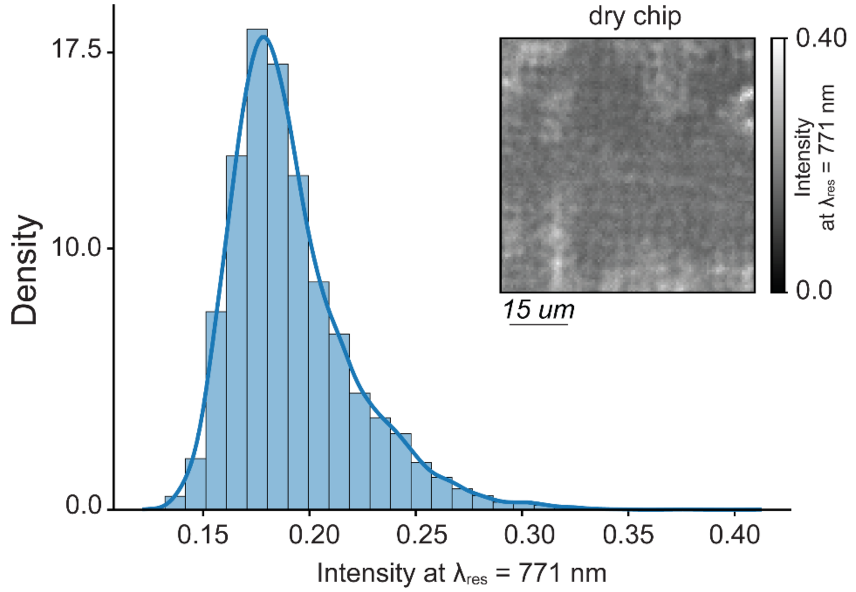

Figure S4. Pixel-level intensity histogram at λ_{res}= 771 nm across 65 x 65 μm^2 metasurface area for a dry chip. Metasurface sensor image captured at λ_{res}= 771 nm.

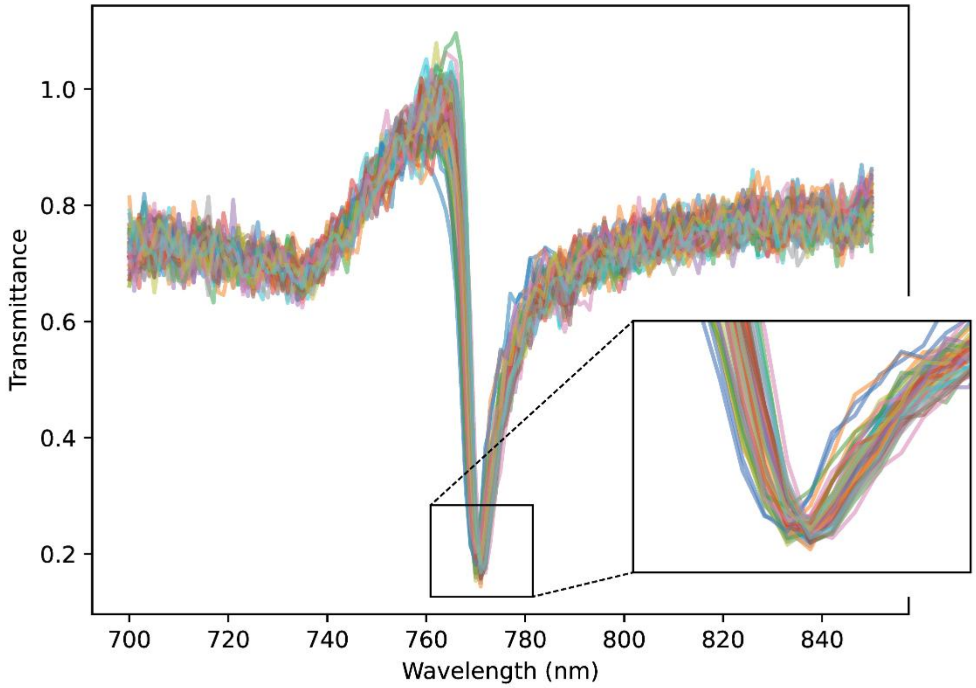

Figure S5. Randomly selected fifty spectra of the qBIC TE mode, centered around λ_{res} ≈ 771 nm from the metasurface in a dry condition and the inset highlighting transmittance variation at λ_{res}.

Note 5. Multiple chips measurements

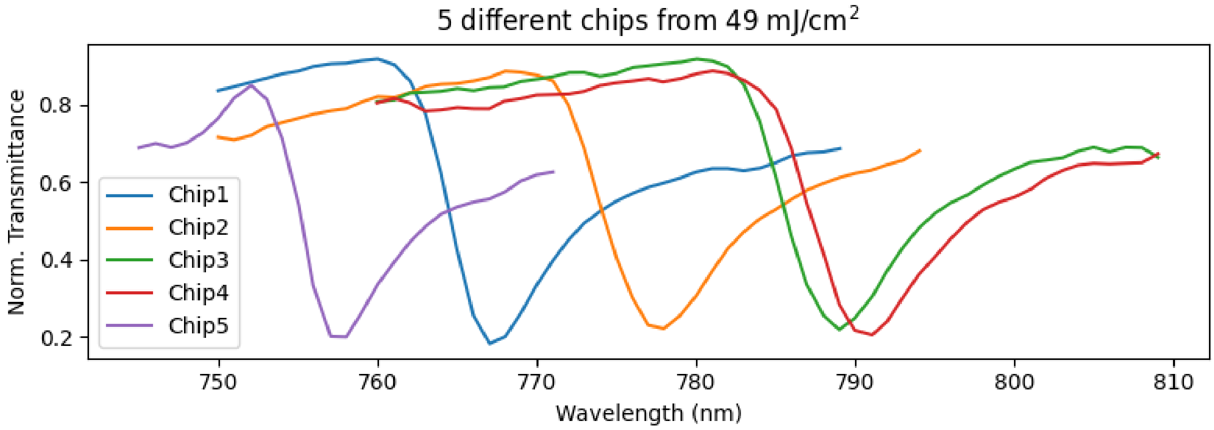

Figure S6. Measured averaged transmission spectra from multiple chips taken from various locations across the same wafer fabricated at the same exposure dose (49 mJ/cm2).

Note 6. Electromagnetic simulation

The transmission spectra and band diagram of the optical resonator were calculated using Tidy3D (Flexcompute, California, USA), a commercial finite-difference time-domain (FDTD) solver. The simulation domain consisted of a unit cell with a central air hole (r_2 = 90 nm) and four surrounding air holes of varying diameters (r_1 = 90–105 nm), patterned on a 160 nm thick Si_3N_4 slab placed on a bulk SiO_2 substrate. All simulations were performed in a vacuum environment, using the optical constants for Si_3N_4[1] and SiO_2[2]. Periodic boundary conditions were applied along the x-, y-, and z-directions. A plane wave with a Gaussian pulse was introduced from above at normal incidence along the z-axis, covering a spectral range of 400–1000 nm. To calculate transmission spectra, a flux monitor was placed beneath the resonator. To evaluate the electric field enhancement, a field monitor was positioned in the x–y plane on top of the resonator at the resonance wavelength. For band diagram calculations in Figure 2c, Bloch periodic boundary conditions were applied by tuning the Bloch vector. A time-domain field monitor was placed inside the resonator to record the electric field response, which was then Fourier-transformed to extract the resonant frequencies supported by the slab at different Bloch vectors. In Figure 2c and S1, the TE and TM modes are excited separately in the simulation by setting the appropriate symmetry conditions with respect to the mirror plane parallel to the slab, even for TE and odd for TM. To obtain the hyperspectral maps in Figure S3, a field monitor was placed below the structure to record the frequency-domain electric and magnetic fields. The spatially resolved Poynting flux density was then calculated at each pixel and normalized to the incident spectrum, yielding transmission as a function of wavelength. The pixel size of the monitor was chosen as 0.65 µm × 0.65 µm at the sample plane to match the experimental imaging condition, and the pixel-wise spectra were stacked into a hyperspectral dataset (x, y, λ) for subsequent analysis.

Note 7. Fabrication of the dielectric resonators

The dielectric device, consisting of periodic nanoscale perforations in a silicon nitride (Si_3N_4) thin film supported by a glass substrate, was fabricated at wafer scale in the Nanofabrication Facility of the University of California, Santa Barbara. A 160 nm thick Si_3N_4 layer was deposited on a 500 nm thick, 4-inch fused silica wafer (University Wafer, Massachusetts, USA) using low-pressure chemical vapor deposition (LPCVD). We used a 248 nm (KrF) deep ultraviolet (DUV) stepper to fabricate the double-hole metasurfaces. This process requires coating the Si_3N_4 layer with a 60 nm bottom anti-reflection layer (BARC), followed by a 230 nm thick DUV chemically amplified photoresist. Hole arrays (diameter: 180–220 nm, period: 510 nm) were patterned using a DUV stepper and a quartz/chromium (Cr) photomask containing multiple design variations. Each wafer patterned with 4 doses per wafer, one in each quadrant, in order to allow detection of any radial variation from subsequent etching steps. Subsequently, DUV resist is developed, and the BARC layer is dry-etched in an O_2 inductively coupled plasma reactive ion etching (ICP-RIE) to clear the holes. Next, exposed Si_3N_4 in the holes was ICP etched *in situ* with a CF_4/O_2 and the remaining DUV resist, and BARC was stripped off. The wafer backside was then etched to remove excess Si_3N_4, and the wafer was diced into 1×1 cm² sensor chips using a resin blade dicing saw (Disco, Tokyo, Japan) with frontside photoresist as protection during both of these steps. The fabricated chips underwent a three-step cleaning protocol before the optical characterization: (1) immersion in MICROPOSIT™ Remover 1165 (Rohm and Haas Electronic Materials, MA, USA) overnight, (2) oxygen plasma treatment (250 W, 1 min, 80 sccm), and (3) the first step of RCA cleaning to ensure a pristine sensor surface.

Note 8. Optical setup and measurement

A broadband SuperK FIANIUM15 laser (NKT Photonics, Birkerød, Denmark) coupled to an LLTF filter (Photon etc., Montreal, Canada) was used as a narrowband light source, providing a full width at half maximum (FWHM) of ~2.5 nm. The sensor was illuminated through a beam collimator and imaged in transmission mode using a 10x objective on the optical path of a TE-200 inverted microscope (Nikon, Tokyo, Japan), equipped with a Prime BSI Express CMOS camera (Teledyne Photometrics, Arizona, USA). Hyperspectral data were acquired over the 400–1000 nm range with a spectral step of 1 nm. The camera exposure time was set to 10 ms. Image acquisition was controlled via a custom MATLAB (MathWorks, Massachusetts, USA) user interface built using the App Designer, which integrated both camera and filter control. For intensity normalization, the light source was imaged through a neutral density (ND) filter with optical density (OD) 3 in the absence of the sensor chip to eliminate the spectral shape of the light source.

Note 9. Data processing

The image data processing was performed using Python libraries in Jupyter Notebook. Q-factors were obtained by fitting the transmittance T with the following Fano fit from [3]:

$$T = \left| ie^{i\varphi} t_0 + \frac{\gamma_r}{\gamma_r + \gamma_a + i(\lambda - \lambda_{res})} \right|^2$$

where $ie^{i\varphi}t_0$ describes the background and the shape of the resonance. The Q-factor can then be calculated as

$$Q = \frac{\lambda_{res}}{2(\gamma_r + \gamma_a)}$$

where $\lambda_{res}, \gamma_r,$ and γ_a are the resonance wavelength, radiative, and absorptive loss rates, respectively.

References

1. K. Luke et al., "Broadband mid-infrared frequency comb generation in a Si_3N_4 microresonator," Opt. Lett. 40(21), 4823–4826 (2015) [doi:10.1364/ol.40.004823].

2. E. D. PALIK, "Handbook of Optical Constants of Solids," Part : Determ. Opt. CONSTANTS, 313–335 (1998) [doi:10.1016/b978-0-08-055630-7.50017-1].

3. S. Fan, W. Suh, and J. D. Joannopoulos, "Temporal coupled-mode theory for the Fano resonance in optical resonators," J. Opt. Soc. Am. A 20(3), 569 (2003) [doi:10.1364/josaa.20.000569].
